# Accuracy of ultrasound performed by medical residents in operating rooms in identifying parathyroid glands in patients with hyperparathyroidism^☆^^☆☆^

**DOI:** 10.1016/j.bjorl.2025.101607

**Published:** 2025-05-28

**Authors:** Regison Rafael Dias Silva, Giovanni Simões de Medeiros, Murilo Catafesta das Neves

**Affiliations:** a Universidade Federal de São Paulo (UNIFESP), São Paulo, SP, Brazil; b Universidade Federal de São Paulo (UNIFESP), Departamento de Otorrinolaringologista e Cirurgia de Cabeça e Pescoço, São Paulo, SP, Brazil

**Keywords:** Ultrasonography, Hyperparathyroidism, Parathyroidectomy

## Abstract

**Objective:**

To describe the findings of US examinations performed by Head and Neck Surgery (HNS) resident physicians in patients in the preoperative period immediately before parathyroidectomy, and to compare these results with those of examinations performed preoperatively (ultrasonography and/ or scintigraphy) and with the findings of surgical procedures.

**Methods:**

Patients in the preoperative period of parathyroidectomy underwent US examinations performed by HNS resident physicians after induction of anesthesia. The findings were registered and later compared with those of preoperative and intraoperative examinations using descriptive statistical analysis and calculation of sensitivity, specificity, and accuracy.

**Results:**

At least one gland was identified in 81% of the patients, most commonly the left inferior parathyroid. There was 63% agreement between the examinations performed in the operating room and the preoperative examinations. Sensitivity of 76%, specificity of 100%, and accuracy of 81.25% were evidenced in identifying parathyroids > 5 mm in patients with hyperparathyroidism associated with Chronic Kidney Disease (CKD), and sensitivity of 33% was found in patients with primary hyperparathyroidism.

**Conclusion:**

US examinations performed by HNS resident physicians in patients preoperatively to parathyroidectomy are accurate (81.25%) in identifying parathyroids compared with intraoperative findings in patients with hyperparathyroidism associated with CKD.

**Level of evidence:**

Level III.

## Highlights

• Ultrasonography is a useful tool for diagnosing head and neck diseases, and recently HN surgeons have incorporated it into their practice.
• US examinations before surgery and during the procedure, just before the parathyroidectomy, were done by the HNS residents.
• These exams accurately identified parathyroids in 81.25% of the patients with hyperparathyroidism with CKD.
• Although this is a small casuistic (11 patients), the method is promising.

## Introduction

Ultrasonography (US) is a radiation-free method that provides dynamic high-resolution images and can be used as the first-line diagnostic tool for various head and neck conditions.^1^

Because of the superficial position of the thyroid and parathyroids in the neck, US is the most sensitive and cost-effective method for their assessment, providing accurate information about their anatomy.^2^

Surgeon-Performed Ultrasound (SPU) is a recent practice, but this tool has been increasingly used in the workup, diagnosis, treatment, and follow-up of a wide variety of diseases of the thyroid, parathyroids, and neck. It is particularly important in assisting with the operative plan, and is not intended to replace the examination performed by radiologists.^3^ Surgeons may apply their practical knowledge of surgical anatomy and parathyroid location (and variability) to the task of ultrasound localization.^3^

However, more than any other imaging modality, US is highly operator- dependent, and adequate training is required to minimize the risk of incorrect results.^4^

Nevertheless, the amount of training needed to obtain sufficient competence in SPU can vary considerably between individual clinicians. Furthermore, little is known about the diagnostic accuracy of SPU currently.^1^, ^5^

## Objectives

• To describe the findings of US examinations performed by HNS resident physicians in patients in the preoperative period immediately before parathyroidectomy;
• To compare these results with those of examinations performed preoperatively (ultrasonography and/or scintigraphy) and with the findings of surgical procedures.

## Method

This is an observational, cross-sectional, descriptive study conducted with patients undergoing Parathyroidectomy for Primary (PHPT) or Secondary (SHPT) hyperparathyroidism associated with Chronic Kidney Disease (CKD) at *Hospital São Paulo* and *Hospital do Rim* between March and May 2021.

Initially, the HNS resident physicians were trained by one of the program preceptors on the functionalities of the US device, as well as on its use aimed at examining the thyroid and parathyroids. To this end, a device identical to those utilized to perform US examinations on patients was used: a ClearVue 350 ‒ Philips equipped with an L12‒4 Hz Active Array probe.

All patients scheduled to undergo parathyroidectomy had their medical records reviewed preoperatively and their clinical data such as diagnosis, previous drug treatment, and preoperative laboratory and imaging tests collected.

In the preoperative period immediately before parathyroidectomy, after induction of anesthesia, the patients were positioned and one of the HNS resident physicians performed an US examination aimed at evaluating the thyroid and parathyroids, observing and describing the presence of changes in the thyroid parenchyma and the position of the visualized parathyroids. During the exam, a scan was performed from the carotid bifurcation to the beginning of the thorax in the craniocaudal direction, and from one carotid to the other in the latero-lateral direction. These data were also registered by the collection instrument.

Intraoperatively, the positions and sizes of the parathyroids and the presence or absence of changes in the thyroid were observed.

The collected data were plotted on an Excel® spreadsheet, their descriptive statistical analysis was performed using percentages, and the sensitivity, specificity, and accuracy of the use of US in the operating room to identify the parathyroids were calculated.

## Results

Eleven patients were examined in the operating room in the preoperative period immediately before parathyroidectomy: three diagnosed with PHPT, one with SHPT, and seven with Tertiary Hyperparathyroidism (THPT). The SPU examination was able to identify the adenoma in only one of the three patients diagnosed with PHPT ‒ a finding equivalent to that of preoperative US, which was also unable to identify the diseased gland in the other two patients. As for the patients diagnosed with SHPT or THPT, it was possible to identify at least one gland in all of them: one parathyroid in two patients, two parathyroids in two patients, and three parathyroids in three patients. The four parathyroids were identified in only one patient. The most commonly identified gland was the left inferior parathyroid, which was identified in eight patients. In total, SPU was able to identify at least one gland in 81% of patients. Table 1 shows the number and distribution of each gland identified in patients with hyperparathyroidism associated with CKD.

**Table 1:** Distribution of glands identified according to anatomical site in patients with hyperparathyroidism associated with CKD.

RSPT	LSPT	RIPT	LIPT
4 (36%)	3 (27%)	5 (45%)	8 (72%)

RSPT, Right Superior Parathyroid; LSPT, Left Superior Parathyroid; RIPT, Right Inferior Parathyroid; LIPT, Left Inferior Parathyroid.

Changes in the thyroid were observed in 5 (45%) patients: two patients with cystic images, two with single nodules, and one with multiple spongiform nodules.

Comparison between the preoperative location examinations and the SPU examinations showed full consistency in seven patients (63%), including the two patients with no glands identified and the only patient with all four glands identified. As for the other patients, three presented correlations of at least one gland identified in the preoperative examinations and only one patient was discordant. Regarding changes in the thyroid, similar findings between the preoperative imaging examinations and the SPU examinations were observed in all patients.

Comparison between the SPU results and the intraoperative findings showed that the largest parathyroid was identified in two of the three patients with only one gland identified. In addition, the largest gland was visualized on US in seven patients.

The left inferior parathyroid, despite being the most frequently identified, was the largest gland in only three patients, and in one of them no gland was identified on US. This may be due to the fact that some examinations may have described as inferior parathyroids those that were actually superior parathyroids.

Table 2 shows the comparison between US and surgical findings, considering parathyroids > 5 mm as increased in size, and Table 3 shows the relationship between the identification of each parathyroid separately with its size observed intraoperatively, both in relation to patients diagnosed with hyperparathyroidism associated with CKD.

**Table 2:** Compararison between preoperative SPU and surgical findings – number of parathyroids identified on US examinations vs. number of parathyroids increased in size.

	RSPT	LSPT	RIPT	LIPT	Total
Number of PT identified on preoperative US	2	1	4	6	13
Number of PT identified on SPU	3	3	5	8	19
Number of PT increased in size identified intraoperatively	6	6	7	8	26

RSPT, Right Superior Parathyroid; LSPT, Left Superior Parathyroid; RIPT, Right Inferior Parathyroid; LIPT, Left Inferior Parathyroid, PT, Parathyroids; US, Ultrasonography; SPU, Surgeon-Performed Ultrasound.

**Table 3:** Relationship between the parathyroid identified on US examination and its intraoperative size observed in patients with hyperparathyroidism associated with CKD.

	PT identified	PT not identified	Total
PT > 5 mm	19	6	25
PT < 5 mm	0	7	7
Total	19	13	32

PT, Parathyroids.

Analysis of these data shows that SPU, in this study, presented sensitivity of 76%, specificity of 100%, and accuracy of 81.25% in identifying parathyroids > 5 mm in patients with hyperparathyroidism associated with CKD.

In patients with PHPT, accuracy of 33% in identifying a diseased gland was observed.

## Discussion

Results of this study showed that at least one parathyroid was identified in patients with SHPT and THPT and in one of the three patients with PHPT, totaling 81% of the patients. These results corroborate the findings by Husen & Kim, who assessed the accuracy of SPU in localizing the parathyroids and identified at least one adenoma in 89% of patients.^6^

The present study also evidenced that the inferior glands were the most commonly identified (61%), as observed by Haber et al., who identified the inferior parathyroids in 73% of patients.^7^ This finding may be related to the fact that the superior parathyroids are located in the tracheoesophageal groove, which hinders their localization on US.

Concerning the size of the parathyroids, those > 5 mm were considered as increased in size, and no glands smaller than that were identified in this study. Stern et al. also reported that there is a relationship between the size and difficulty in identifying a gland on US, with smaller accuracy for parathyroids < 1 cm (24% compared to 75% for glands > 1 cm).^8^

The present study also found that SPU presented sensitivity of 76%, specificity of 100%, and accuracy of 81.25% in identifying diseased parathyroids in patients with hyperparathyroidism associated with CKD. Few studies have evaluated the accuracy of US in these patients. Purcell et al. observed sensitivity of 33% and specificity of 100%.^9^

Adenomas were identified in one of the three PHPT patients, showing sensitivity of 33%, which is lower than that reported in other studies that assessed these patients. Purcell et al. observed sensitivity and specificity of 66 and 98%, respectively, for PHPT patients,^9^ whereas Stern et al. found sensitivity of 76.2%.^8^ The low sensitivity found may be related to the small number of patients examined because, due to the COVID-19 pandemic, the number of PHPT surgeries was reduced in the Institution where this study was carried out. In addition, preoperative US examinations performed by radiologists were also unable to identify the diseased gland in the two patients, which suggests a greater technical difficulty in examining them.

The small sample size is a limitation to this study, which hindered more robust statistical evaluations. There is intention to continue with this study, evaluating a larger number of patients in order to reach more consistent conclusions.

## Conclusion

Ultrasonography examinations performed by HNS resident physicians in patients preoperatively to parathyroidectomy are accurate (81.25%) in identifying parathyroids compared with intraoperative findings in patients with hyperparathyroidism associated with CKD.

## Funding

The study was carried out with financial support from the researchers themselves.

## Declaration of competing interest

The authors declare no conflicts of interest.
